# Percutaneous gastrostomies in advanced cancer

**DOI:** 10.1097/j.pbj.0000000000000238

**Published:** 2023-12-13

**Authors:** José António Ferraz-Gonçalves, Susana Amaral, Filipa Pereira, Lígia Rodrigues Santos, José Guilherme Assis, Sérgio Alves, Diana Martins

**Affiliations:** a Faculdade de Medicina da Universidade do Porto, Porto, Portugal; b Centro Hospitalar de Vila Nova de Gaia e Espinho, Vila Nova de Gaia, Portugal; c Hospital de Braga, Braga, Portugal; d Centro Hospitalar de Trás-os-Montes e Alto Douro, Vila Real, Portugal; e Unidade Local de Saúde do Nordeste, Bragança, Portugal

**Keywords:** gastrostomies, advanced cancer, dysphagia, head and neck cancer, esophageal cancer

## Abstract

**Introduction:**

Gastrostomies can be performed percutaneously by interventional radiology (PRG) or endoscopy (PEG).

**Methods:**

Retrospective analysis of patients with advanced cancer who underwent a gastrostomy in 2017 in an oncology center.

**Results:**

In 2017, 164 patients underwent gastrostomies, and 137 (84%) were male. The median age was 60 years (range: 38–91). The predominant Eastern Cooperative Oncology Group (ECOG) performance status stage was 1, with 73 (45%) patients. Head and neck cancer was the most common diagnosis, with 127 (77%) cases. The most frequent reason for performing a gastrostomy was dysphagia, 132 (81%). Most gastrostomies were PEG, 121 (74%), followed by PRG, 41 (25%), and surgery, 2 (1%). Early complications occurred in 86 (52%) patients, and the most frequent of them were local pain in 69 (80%) patients and minor local bleeding in 13 (15%). Late complications occurred in 90 (55%) patients, and the most frequent was also local pain in 57 (63%) patients, followed by local infection in 8 (9%), tube extrusion in 7 (8%), and stomal leakage in 7 (8%). In the multivariable analysis, the factors associated with survival were lymph node metastases and the ECOG performance status. Until June 30th, 2022, 123 (75%) patients had died, and 41 (25%) were still alive.

**Conclusion:**

Gastrostomies were performed predominantly in ECOG performance stage 1 patients with head and neck cancer and symptoms of dysphagia, and PEG was the most common procedure.

## Introduction

Percutaneous gastrostomies in patients with advanced cancer aim to provide food and fluids when limited by functional or mechanical dysphagia. In addition, when gastrostomies are in place, they can also be used to administer medication. Venting gastrostomies can also decompress the stomach and bowel in some patients with bowel obstruction.^1^

Dysphagia is common in patients with head and neck and esophageal cancers. Dysphagia is often due to the strategic location of those cancers and not necessarily to their advanced stages. The starvation caused by dysphagia can quickly deteriorate the patient's state. These cases differ significantly from the deterioration associated with anorexia in advanced stages when artificial feeding is not indicated. Therefore, patients with an expected life of >1 month who cannot eat should be considered for enteral nutrition^2^ because, with no intake, they may starve to death and not die from underlying cancer itself. Besides obstructive dysphagia, other situations can justify the performance of a gastrostomy, such as orocutaneous and tracheoesophageal fistulas. Gastrostomies may also be performed prophylactically when mucositis preventing adequate nutrition induced by radiotherapy head and neck tumors is foreseen.^3^

The Norwegian surgeon Egberg, in 1837, conceived a surgical technique to feed patients by a tube placed in the stomach through the abdominal wall.^4^ Charles Sedillot, from Strasburg, performed the first surgery of this type, a surgical gastrostomy, in 1849.^4^ All the gastrostomies recorded since then resulted in patient death, usually from peritonitis, and only in 1876, in Paris, did Verneuil perform the first successful surgical gastrostomy.^4^ Many years had passed before a new technique was introduced: In 1979, the North Americans Gauderer and Ponsky performed the first percutaneous endoscopic gastrostomy (PEG).^5^ In 1981, the Canadian Preshaw introduced percutaneous radiological gastrostomy (PRG).^6^

Nasogastric tubes are also a possibility to overcome dysphagia. It is a method whose execution is more accessible and cheaper; therefore, it is more extensively used. However, it is usually seen as a technique that should be reserved for short-term feeding. Although without unanimity, a 6-week period divides short-term from long-term enteral feeding.^7^ Long-term nasogastric tubes may cause several complications, such as pulmonary aspiration of infused solutions, lesions of the gastrointestinal tract mucosa by the tip of the tube, ear and nose infections, esophageal strictures, and vocal cord and pharyngeal paralysis.^7^ Besides those complications, there is a self-image problem with the nasogastric tubes.

Gastrostomies are not exempt from complications, but most are associated with the insertion technique rather than prolonged use.^7^ A Cochrane review reported no significantly different complication rates between surgical feeding ostomies and natural orifice feeding tubes.^8^ However, PEG can be associated with severe and life-threatening complications, such as sepsis, peritonitis, hemorrhage, tube migration perforation, and buried bumper syndrome.^9^ A recent study concluded that surgical feeding ostomies were associated with more serious adverse events than natural orifice feeding tubes.^10^

Some years ago, we published a study on gastrostomies in palliative care.^11^ At that time, almost all gastrostomies were performed by interventional radiology. However, owing to organizational changes and higher availability, most are currently performed by endoscopy, although many are yet performed by radiology. Owing to that change, a retrospective reassessment was performed to determine the indications, complications, and possible differences between both techniques. Possible factors associated with survival were also analyzed.

## Methods

This is a descriptive retrospective study of patients with advanced cancer who underwent a gastrostomy in 2017 (from January 1st to December 31st) in an oncology center, identified through the center's electronic records. Advanced cancer was defined according to the National Cancer Institute Dictionary of Cancer Terms (“cancer that is unlikely to be cured or controlled with treatment. Cancer may have spread from where it first started to nearby tissue, lymph nodes, or distant parts of the body. Treatment may be given to help shrink the tumor, slow the growth of cancer cells, or relieve symptoms”).^12^ All patients with the conditions mentioned above were included; no patients were excluded.

For this study, the researchers collected demographic data, the performance status, diagnosis, metastases, the reason for performing the gastrostomy, the presence of tracheostomies, the method used for inserting the gastrostomy, complications, and mortality.

To analyze the complications of gastrostomies, we divided them according to early and late when its occurrence was in the first 5 days after the procedure or after 5 days. Our previous study used the same classification with the same rationale: to distinguish complications related to the procedure from those due to continuous tube use.

For statistical purposes, data were analyzed to identify coding errors and inconsistencies, and corrections were made where needed. An exploratory analysis of the data was performed to describe the sample. The comparison of characteristics between PEG and PRG was performed using the chi-square test. The Cox regression was used for univariable and multivariable analysis of survival, and a confidence interval of 95% was calculated. The level of significance was deemed to be 0.05. Data were analyzed using the software IBM Corp, released 2020, IBM SPSS Statistics for Windows, version 27.0, Armonk, NY: IBM Corp.

The study was authorized by the Ethics Committee of the hospital (CES. 284/021).

## Results

During the study period, 164 patients underwent gastrostomies, and 137 (84%) were male. The median age was 60 years (range: 38–91). The predominant Eastern Cooperative Oncology Group (ECOG) performance status stage was 1, with 73 (45%) patients, followed by stage 2, 51 (31%). Head and neck cancer was diagnosed in 127 (77%) cases, followed by esophageal cancer in 26 (16%) (Table 1). Locally advanced cancers occurred in 140 (85%) patients, and 59 (36%) had lymph node metastases. The most frequent indication for performing a gastrostomy was dysphagia, 129 (79%), followed by prophylactic in 12 (7%) (Table 1). PEGs were 121 (74%), PRG 41 (25%), and surgery 2 (1%). Surgery was only used when other techniques could not be performed. There were no significant differences concerning sex (*P* = .820), age (*P* = .554), performance status (*P* = .157), or diagnoses (*P* = .653) of patients between those who underwent gastrostomies performed by endoscopy or radiology.

**Table 1.** Demographic and clinical data

Sex, n (%)	
Male	137 (84)
Female	27 (16)
Primary cancer, n (%)	
Head and neck	127 (77)
Esophagus	26 (16)
Head and neck + esophagus	2 (1)
Other	9 (6)
Metastases, n (%)	
Locally advanced	140 (85)
Lymph nodes	59 (36)
Lung	15 (9)
Bone	17 (10)
Liver	4 (2)
CNS	3 (2)
Peritoneum	2 (1)
Tracheostomy, n (%)	
Yes	55 (34)
No	109 (66)
ECOG, n (%)	
0	7 (4)
1	73 (45)
2	51 (31)
3	30 (18)
4	3 (2)
Motive, n (%)	
Dysphagia	129 (79)
Prophylactic	12 (7)
Orocutaneous fistula	4 (2)
Esophageal cutaneous fistula	4 (2)
Tracheoesophageal fistula	4 (2)
Other	11 (7)
Method, n (%)	
Endoscopy	121 (74)
Radiology	41 (25)
Surgery	2 (1)
Age, median	60 years (range: 38–91)

CNS, central nervous system.

Early complications occurred in 86 (52%) patients, and the most frequent of them were local pain in 69 (80%) patients and minor local hemorrhage in 13 (15%). Late complications occurred in 90 (55%) patients, and the most frequent was also local pain in 57 (63%) patients, followed by local infection in 8 (9%), tube extrusion in 7 (8%), and stomal leakage in 7 (8%) (Table 2). There were no significant differences between the radiological and endoscopic methods concerning early (*P* = .295), late (*P*= .217), or total (*P* = .166) complications.

**Table 2.** Complications

Early, n (%)	
Pain	69 (80)
Bleeding, local	13 (15)
Nausea/vomiting	3 (3)
Stomal leak	1 (1)
Total	86* (100)
Late, n (%)	
Pain	57 (63)
Infection, local	8 (9)
Tube extrusion	7 (8)
Stomal leakage	7 (8)
Bleeding	4 (4)
Tube blockage	1 (1)
Extrusion + leakage	1 (1)
Infection, local + pain	1 (1)
Leakage + pain	1 (1)
Leakage + nausea/vomiting	1 (1)
Leakage + infection, local	1 (1)
Extrusion + infection, local	1 (1)
Total	90† (100)

* 52% of the total.

† 55% of the total.

In univariable analysis, the factors associated with survival were lymph node metastases, locally advanced cancer, ECOG performance status, the method used for gastrostomy, and the motive for the gastrostomy performance (Table 3). On the contrary, sex, age, diagnosis, and the presence of a tracheostomy were not. In the multivariable analysis, lymph node metastases and an ECOG performance status >1 were associated with a poorer prognosis (Table 4).

**Table 3.** Univariable analysis of factors influencing survival

Factors	HR	95% CI		*P*
		Lower	Upper	
Age				
60 years and younger	1			
Older than 60 years	1.24	0.87	1.77	.235
Sex				
Male	1			
Female	1.06	0.66	1.72	.803
Primary cancer				
Head and neck	1			
Other	1.37	0.91	2.07	.128
Tracheostomy				
No	1			
Yes	0.92	0.63	1.34	.662
Locally advanced				
No	1			
Yes	1.98	1.11	3.52	**.020**
Lymph node metastases				
No	1			
Yes	2.32	1.62	3.33	**<.001**
Method				
Radiology	1			
Endoscopy	0.66	0.45	0.99	**.043**
ECOG				
0–1	1			
2	1.76	1.12	2.67	**.007**
3–4	4.63	2.29	7.37	**<.001**
Motive				
Other	1			
Dysphagia	1.01	0.61	1.64	.990
Prophylactic	0.24	0.08	0.71	**.010**

*P*-value from Cox regression.

Bold - statistically significant.

CI, confidence interval; HR, hazard ratio.

**Table 4.** Multivariable analysis of factors associated with survival

	HR	95.0% CI		*P*
		Lower	Upper	
Lymph node metastases				
No	1			
Yes	2.11	1.46	3.05	**<.001**
ECOG				
0–1	1			
2	1.68	1.10	2.56	**.016**
3–4	4.61	2.87	7.39	**<.001**

*P*-value from Cox regression.

Bold - statistically significant.

CI, confidence interval; HR, hazard ratio.

Until June 30th, 2022, 123 (75%) patients had died, and 41 (25%) were still alive: 29 (23%) of those who underwent a gastrostomy due to dysphagia, 8 (67%) due to prophylaxis, and 4 (17%) for other motives. Admission to the palliative care service of the center occurred in 38 (23%) patients. All 12 patients who underwent prophylactic gastrostomy had it removed because of no evidence of disease after chemotherapy and radiotherapy. Of those patients, 10 had head and neck cancer, and two had esophageal cancer; all the eight still alive had head and neck cancers.

## Discussion

This study focuses on neoplastic diseases; the most frequent cancer type was head and neck, followed by esophagus cancer. In those cancers, the inability to eat may not result from a late phase of the disease but from the tumor's location. Therefore, if the obstruction can be bypassed, the nutritional status may be maintained, and the prognosis may change dramatically because without food and mainly without liquids life is predictably very short.^2^ As occurred in this study, some patients can be cured or at least have the disease controlled, allowing the gastrostomy removal.

In this study, male patients predominate: 84% were male patients, like in other studies of gastrostomies in advanced cancer, as examples are 78% in head and neck patients^13^ and 78% in patients with esophageal cancer.^14^ In addition, in our previous study on gastrostomies in advanced cancer, 80% were male. The higher incidence of those cancers in men is attributed to also higher smoking and alcohol use. Cancer incidence is higher in male patients at most shared sites, and a recently published study found that this predominance is mainly independent of risk factors, underlining the role of sex-related biologic factors.^15^ However, the male effect was statistically insignificant for some cancer sites, including head and neck and esophagus-squamous cell carcinoma.^15^

Most patients had at least a good performance status as assessed by the ECOG scale, suggesting, in general, a good selection of patients for gastrostomies. There were, however, a few patients with ECOG 4. Some patients responded well to the treatment, and the gastrostomies could be removed, as was described before.^9,16^ As in this study, gastrostomies are prophylactically used before treatment, sometimes in patients without dysphagia, to avoid malnutrition caused by the toxic effects of radiotherapy and chemotherapy, with the intention of its remotion later.^3^

Complications may occur associated with the insertion of the gastrostomy tube. The most frequent was local pain related to the procedure. Later more severe complications occurred, such as leakage of the gastric content, tube extrusion, tube blockage, and local infection, which were like other studies' complications.^9,17^ However, contrary to the previous study, where one death occurred due to peritonitis,^11^ no deaths related to the procedure occurred. Peritonitis is a severe complication described in other studies that may be a significant cause of death.^17^

Most gastrostomies were performed by endoscopy, although one-quarter were performed by interventional radiology. On the contrary, in the previous study, 96% of the feeding tubes were inserted by interventional radiology, reflecting the change in the practice of the hospital. There were no significant differences between the two techniques regarding complications and survival. The comparative studies of the two techniques show minor differences in safety between them,^18,19^ although PEG was 44% more expensive in one study. However, in a study comparing both methods that included more than 33,000 patients, PEG was associated with a lower incidence of adverse outcomes and 30-day mortality than the radiological method.^20^

In this study, the factors associated with survival were the presence of lymph node metastases and the performance status reflecting the disease's advanced stage and the patient's physical shape. Other metastases also could have an influence, but their prevalence was too low.

This study has some limitations related to its retrospective nature and its limitation to one hospital. Owing to its retrospective nature, it is impossible to capture data accurately related to the quality of life. Those data would be critical in assessing the worthiness of gastrostomies in patients with advanced cancer.

## Conclusion

Gastrostomies are essential to support the life of some patients with advanced cancer that prevents swallowing, mainly head and neck and esophageal cancers. However, complications are frequent. Patient selection is essential because survival is related to the disease's stage and the patient's fitness. The method used to perform percutaneous gastrostomy does not influence the results. Therefore, there is no need to change the method usually used in each center.

## Conflict of interest

The authors declare no conflicts of interest.

## Author Contributions

J.A.F.-G. proposed the study and wrote the first draft of the manuscript. All other authors collected data, reviewed the manuscript, and contributed with their comments and suggestions for the final version. All authors approved the final version.

## Funding

There was no funding for this study.
